# Pyroptosis: A new insight of non-small-cell lung cancer treatment

**DOI:** 10.3389/fonc.2022.1013544

**Published:** 2022-11-29

**Authors:** Xi Chen, Jianzhang Wu, Jiabing Wang

**Affiliations:** ^1^ School of Medical College, Taizhou University, Taizhou, Jiaojiang, Zhejiang, China; ^2^ School of Pharmaceutical Sciences, Wenzhou Medical University, Wenzhou, China; ^3^ Department of Pharmacy, Municipal Hospital Affiliated to Taizhou University, Taizhou, China

**Keywords:** pyroptosis, NSCLC, programmed cell death, prognosis, therapy

## Abstract

Non-small cell lung cancer (NSCLC) has become one of the most common malignant tumors. Emerging evidence has shown that tumor resistance to apoptosis by damaging or bypassing apoptotic cell death is a major contributor to poor responses to therapy in patients with NSCLC. Pyroptosis is a new type of cytolytic and inflammatory programmed death distinct from apoptosis. Currently, pyroptosis has been reported to cause a strong inflammatory response and significant tumor suppression. It is considered a promising therapeutic strategy and prognosis for NSCLC. In this review, we summarized the characteristics of pyroptosis from its underlying basis and role in NSCLC, thereby providing the potential of pyroptosis as a therapeutic strategy and highlighting the challenges of activating pyroptosis in NSCLC treatment.

## 1 Introduction

Globally, non-small cell lung cancer (NSCLC) accounts for 85% of common lung cancer types and is mainly divided into lung adenocarcinoma (LUAD) and lung squamous cell carcinoma (LUSC) (1). The efficacy of NSCLC treatment largely depends on the stage of the disease in which the patient is diagnosed. Adjuvant therapy can prolong postoperative survival, but only a small number of patients with NSCLC have resectable tumors. Over the past two decades have been witnessed that molecular targeted therapies and immunotherapies for NSCLC with markedly improved outcomes (1). However, the majority of advanced NSCLC patients develop resistance to apoptosis under these treatments and eventually progress and death (2). Thus, for patients with locally advanced or metastatic disease, the prognosis and 5-year survival rates remain extraordinarily poor (3). Through bypassing apoptosis or activating non-apoptotic programmed cell death in NSCLC has attracted great attention and is crucial for the development of new therapeutic approaches.

**Figure 1:**
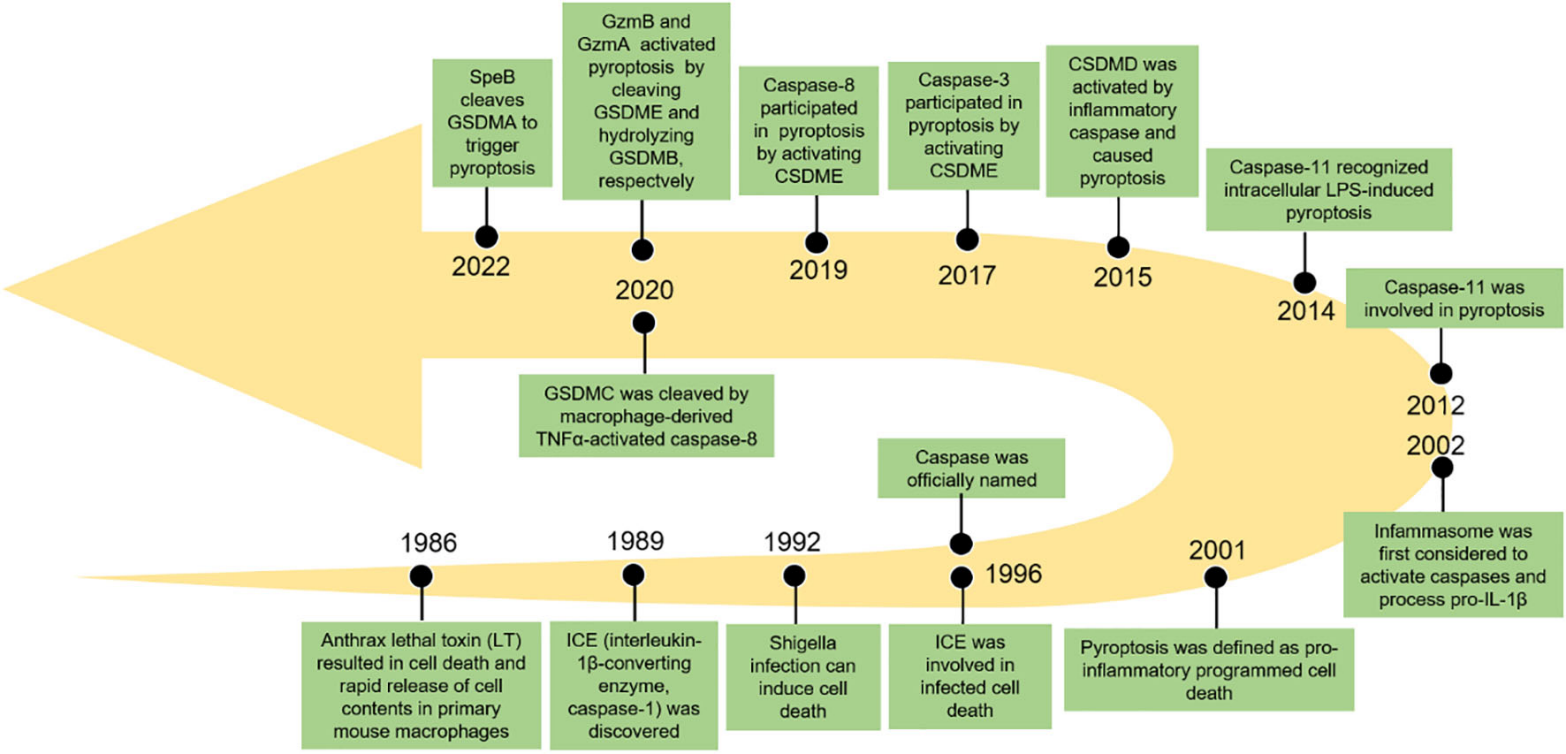
The key events in the history of pyroptosis.

**Figure 2:**
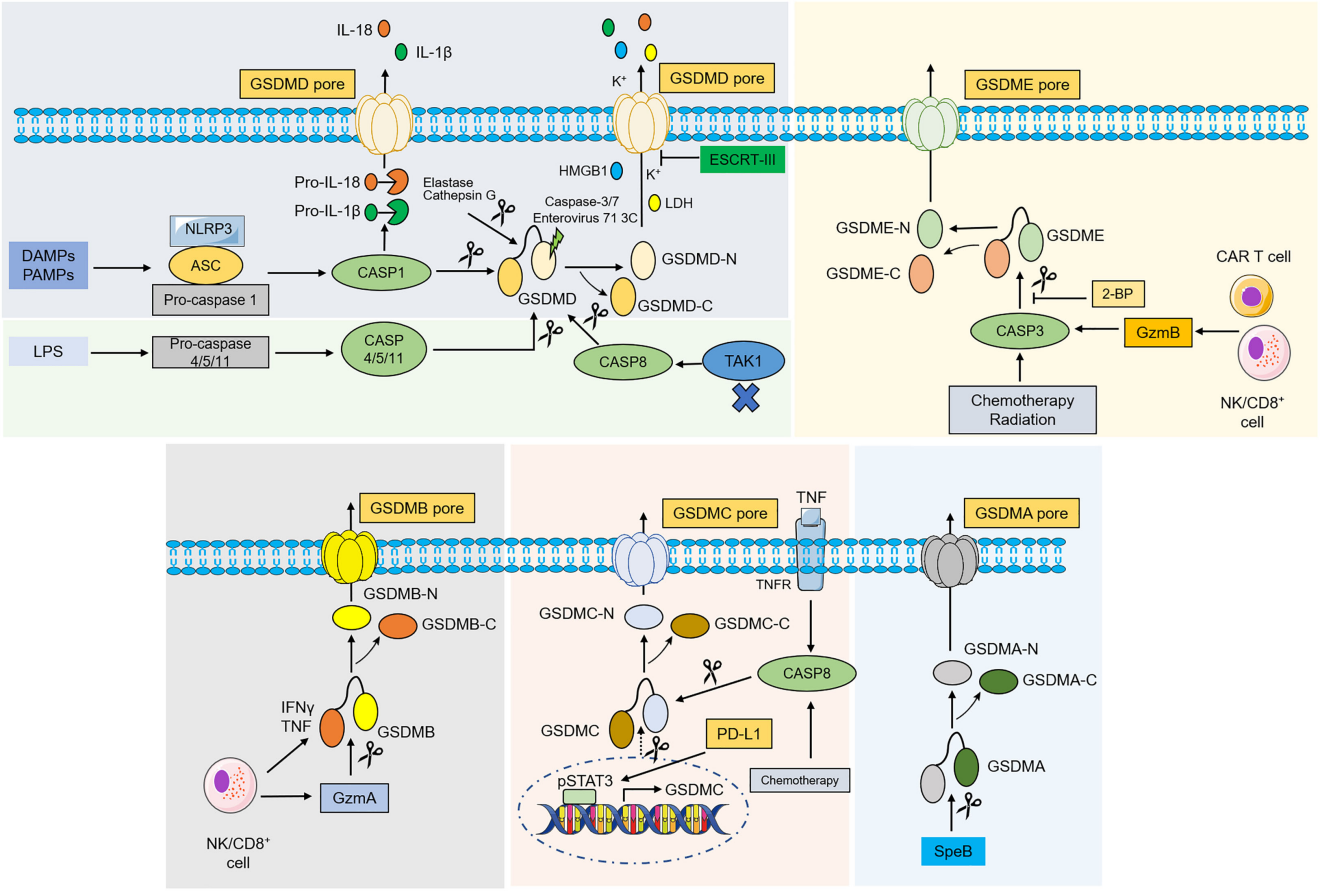
Molecular mechanism of gasdermins-mediated pyroptosis. In classical pathway, PAMPs and DAMPs are recognized by various stimuli and then activate conformational change of the NLRP3 inflammasome, and NLRP3 recruits ASC and binds to pro-caspase-1 to form NLRP3- ASC-pro-caspase-1 protein complex, which then cleaves pro-caspase-1 into caspase-1. Activated caspase-1 destroys the intramolecular auto-inhibitory structure of GSDMD by cleaving GSDMD, which promotes GSDMD-NT generation and induces pyroptosis. Caspase-1 can also recognize and catalyze pro-IL-1β, pro-IL-18, leading to maturation of IL-1β, IL-18. In the noncanonical pathway, caspase-4/5/11 are recognized by LPS from Gram-negative bacteria in the cytoplasm, which then lead to pyroptosis by cleaving GSDMD. Inhibiting TAK1 can stimulate the activation of caspase-8, which cleaves GSDMD and causes pyroptosis. An elastase and cathepsin G can also cleave GSDMD and activates pyroptosis. ESCRT-III complex repairs the membrane by removing the GSDMD pore through exocytosis. Caspase-3, caspase-7 and enterovirus 71 3C cleave the GSDMD and generate a nonfunctional N-terminus. Under the action of chemotherapy, radiation and GzmB, caspase-3 was activated and cleaves GSDME to liberate the cytotoxic N-GSDME, inducing pyroptosis. 2-BP can reverse caspase-3/GSDME-mediated pyroptosis. In addition, GSDMB is cleaved by GzmA, while under hypoxia conditions, GSDMC is cleaved by TNFα-activated-caspase-8 and transcriptionally upregulated through pSTAT3 interaction with PD-L1. SpeB directly activates GSDMA and induce pyroptosis. Abbreviations: DAMPs, danger-associated molecular patterns; PAMPs, pathogen associated molecular patterns; ASC, apoptosis-associated speck-like protein contain a CARD; LPS, lipopolysaccharides; NLRP3, NLR family pyrin domain-containing 3; TAK1, transforming growth factor beta-activated kinase 1; LDH, lactate dehydrogenase; HMGB1, high-mobility group box 1; IL, interleukin; TAK1, transforming growth factor beta-activated kinase 1. ESCRT, endosomal sorting complex required for transport; GzmA/B, granzyme A/B; 2-BP, 2-bromopalmitate; SpeB, streptococcal pyrogenic exotoxin B; pSTAT3, phospho-signal transducer and activator of transcription 3; PD-L1, programmed death-ligand 1.

**Figure 3:**
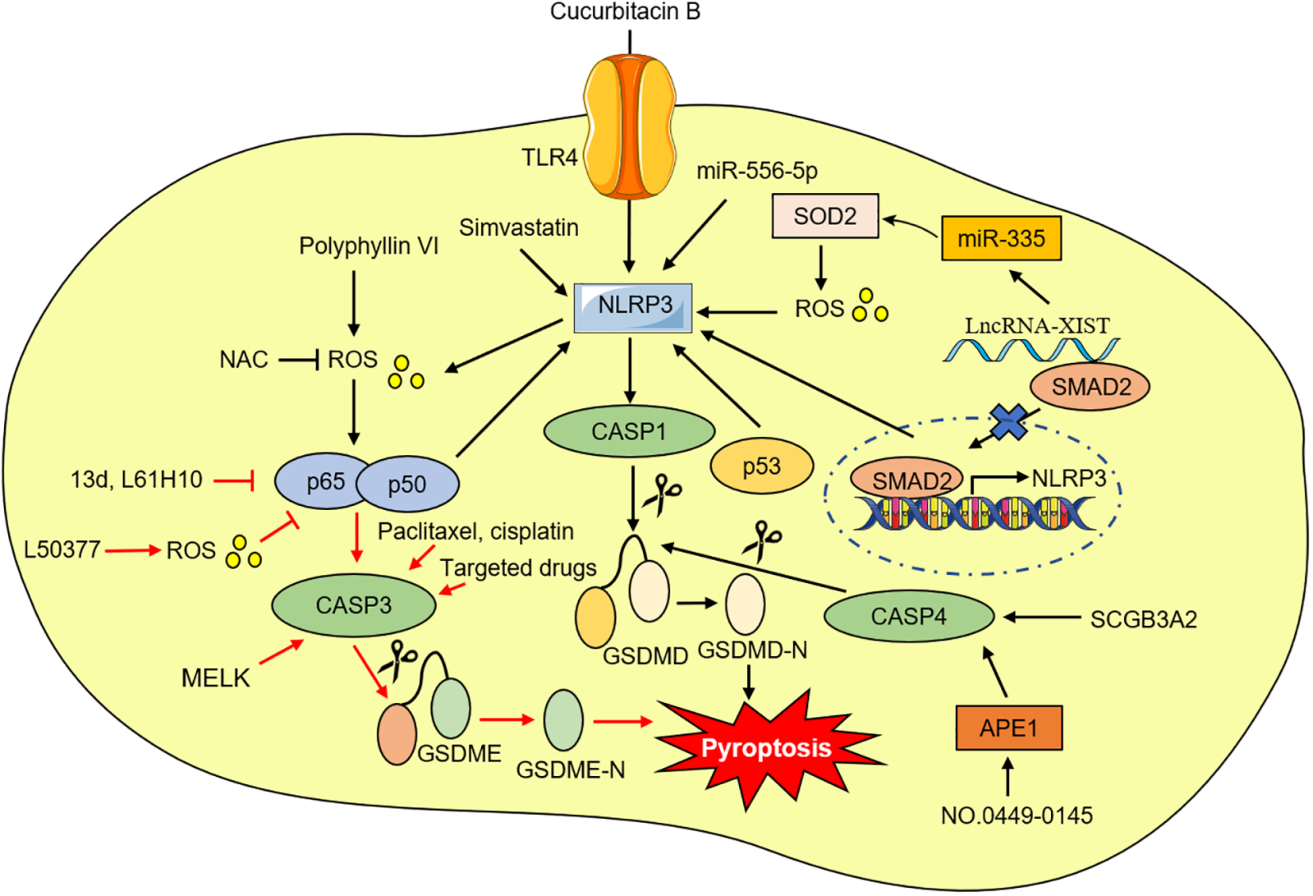
The possible mechanism of targeting pyroptosis in NSCLC.

Pyroptosis, also known as inflammatory necrosis, is a new non-apoptotic programmed cell death mediated by inflammatory responses, and is related to many diseases, such as infectious, spontaneous inflammation, and autoimmune conditions (4). Since the discovery in 1986 that anthrax lethal toxin (LT) caused cell death and rapid release of cellular contents in primary mouse macrophages (5), researchers have discovered caspase families and characterized and explained their function preliminarily over the next 15 years (6–8), while pyroptosis was discovered in 1992 and the term pyroptosis was coined in 2001 (9, 10). It was first hypothesized in 2002 that inflammasomes activate inflammatory caspases and process pro-IL-1β (11), and mouse caspase-11 can trigger non-classical inflammasome activation. It was later established in 2012 that gram-negative bacteria can utilize lipopolysaccharide (LPS) to activate caspase-11 intracellularly. This activation is independent of the traditional LPS extracellular receptor toll-like receptor 4 (TLR4) (12). It was also been reported that caspase-1 or caspase-11/4/5 in the activated state can cleave gasdermin D (GSDMD) to form GSDMD-N-terminal (GSDMD-NT) and GSDMD-C-terminal (GSDMD-CT). Moreover, the GSDMD-CT can oligomerize to form pores and induce pyroptosis (13). In 2017, the results of Wang et al. and Rogers et al. discovered that conventional chemotherapeutic drugs are able to cause cell death *via* pyroptosis when tumor-expressing gasdermin E (GSDME) is cleaved by activated caspase-3 to form GSDME-N-terminal (GSDME-NT) and GSDME-C-terminal (GSDME-CT) in various cancers. The membrane perforates and releases interleukin-1β (IL-1β), interleukin-18 (IL-18), and cellular contents into the extracellular space and activates inflammatory response (14, 15). In 2020, it was reported that granzyme B (GzmB) can activate pyroptosis by cleaving GSDME, which stimulates antitumor immune responses and further inhibits tumor growth (16). Additionally, it was found that granzyme A (GzmA) is released from cytotoxic lymphocytes and then enters target cells through perforin, where it causes cell pyroptosis by hydrolyzing GSDMB in the same year (17). Quite recently, streptococcal pyrogenic exotoxin B (SpeB) was found to trigger keratinocyte pyroptosis by cleaving GSDMA after Gln246, resulting in the release of an active N-terminal fragment that induces pyroptosis. This renewed our understanding of pyroptosis (18) (**Figure 1**). The discovery that pyroptosis in cancers eliminates apoptosis- or necroptosis-resistant cancer cells has also advanced our understanding of cancer and provided new ideas for its management. Significant progress has also been made in identifying the underlying mechanisms and prognosis of pyroptosis in NSCLC (**Figures 2** and **3**). Moreover, lung cancer cells tend to trigger an autoimmune response, which sets off a series of inflammatory cascades. Therefore, targeting pyroptosis can provide a new and promising approach to treating lung cancer. To provide potential perspectives for the treatment of NSCLC, we for the first time primarily summarized the between cardinal mechanisms and role of pyroptosis and NSCLC in this review.

## 2 The molecular mechanism of pyroptosis

### 2.1 The canonical pathway

The canonical pyroptotic death is dependent on caspase-1 and inflammasome assembly and is accompanied by GSDMD cleavage and release of IL-1β and IL-18 (13, 19–21). Inflammasomes are multi-protein complexes comprised of sensor proteins, apoptosis-associated speck-like protein containing a C-terminal caspase recruitment domain (ASC), and pro-caspase-1 that can assemble in response to microbial infections, pathogen-associated molecular patterns (PAMPs) (e.g., lipoteichoicacid, peptidoglycan, glycans, and LPS), and damage-associated molecular patterns (DAMPs) (e.g., high mobility group box 1, heat shock proteins, and DNA) (22). Upon intracellular encounter with various risk factors, a range of intracellular sensor proteins, such as the nucleotide-binding oligomerization domain (NOD)-like receptor (NLR) family (e.g., NLRP3, NLRP1, NLRC4), absent in melanoma 2 (AIM2), or pyrin proteins, homooligomerize and recruit the ASC, bridging inflammasome sensors with pro-caspase-1 and leading to mature caspase-1 activation through self-cleavage (22). The GSDMD was discovered as a potent pyroptosis executioner for caspase-1 and mouse caspase-11 or human caspase-4 and 5 (19–22). The pro-inflammatory cytokines IL-1β and IL-18 are matured under the activation of caspase-1 and cleave GSDMD after a conserved Asp275 residue (Asp276 residue for mouse GSDMD) in the flexible peptide linker to form GSDMD-NT and GSDMD-CT, and further form GSDMD pore on plasma membrane through the liberated GSDMD-NT (23, 24). The IL-1β and IL-18 are released into the extracellular milieu through the GSDMD pore. This facilitates immune cell infiltration and establishes an inflammatory microenvironment (25). In addition, excessive GSDMD pore formation enables the release of other pro-inflammatory contents, including high-mobility group box 1 (HMGB1) and lactate dehydrogenase (LDH), thereby exacerbating inflammatory responses and recruiting immune cells (26, 27). It is worth noting that cells may release IL-1β and IL-18 after inflammasome activation without cell death. However, the exact mechanism is unclear (28).

### 2.2 The non-canonical pathway

Unlike the canonical inflammasome pathway, the non-canonical pathway relies on caspase-4/5/11. These pro-caspases can be activated by direct binding of the caspase activation and recruitment domain (CARD) to intracellular LPS, which contributes to stimulate caspase-mediated cleavage of GSDMD and ultimately causes pyroptosis (29, 30). However, caspases-4/5/11 are unable to cleave pro-IL-1β/pro-IL-18 but can mediate the efflux of potassium ions and the maturation and secretion of IL-1β/IL-18 *via* the NLRP3/caspase-1 pathway (31). Recent studies have shown that a p10 product generated from specific autocleavage at Asp289/Asp285/Asp316 in caspase-4/11/1, respectively, is necessary for GSDMD cleavage and pyroptosis induction (32). In addition to caspase-1/4/5/11, GSDMD can be cleaved by caspase-8 during Yersinia infection which can inhibit the transforming growth factor-β (TGF-β)-activated kinase 1 (TAK1) in mouse macrophages (33, 34). Moreover, an elastase released from cytoplasmic granules and cathepsin G can directly induce pyroptosis of neutrophils and monocytes, respectively, in a GSDMD-dependent manner (35).

Several other negative regulations of pyroptosis pathways have also been reported. In monocytes and macrophages, active apoptotic caspases-3 and 7 were found to inactivate GSDMD by cleaving GSDMD at the Asp87 residue, which in turn induces apoptosis, but not pyroptosis (36). Enterovirus A71 (EV-A71) is one of the major pathogens causing hand-foot-and-mouth disease (HFMD) in infants and young children (37). It was found that EV-A71 can cleave GSDMD at the Gln193-Gly194 residue using utilize a 3C protease, resulting in a truncated GSDMD-NT lacking pore-forming capabilities, residues 1-193, and inhibition of pyroptosis (38). The generation of GSDMD pores is the main cellular mechanism for the release of inflammatory molecules and cell pyroptosis. Preventing or prolonging the process of pyroptosis through repairing GSDMD pores may be another promising approach to inhibiting pyroptosis. The endosomal sorting complex required for transport (ESCRT), a peripheral membrane protein complex, that plays an important role in repairing cell membrane damage (39). In the ESCRT system, ESCRT-III was found to preserve the integrity of the cytoplasmic membrane to restrain pyroptosis (40). Mechanistically, calcium ions enter the cells through GSDMD pores as signaling molecules and launch membrane repair by enrolling the ESCRT-III machinery into damaged membrane regions (40).

### 2.3 The caspase-3-mediated pathways

The cytosol contains a group of structurally related cysteine proteases known as caspases, all of which can specifically break peptide bonds after aspartic acid residues (41, 42). Caspase-8 and caspase-3 are originally defined as the promoter and effector of apoptotic caspases in the apoptosis process, respectively (43, 44). However, it has been discovered recently that the activation of caspase-3 or caspase-8 is not specific to apoptosis. Caspase-3 after chemotherapeutic drug activation cleaves human GSDME at position 270 amino acid residue to produce GSDME-NT in tumor cells with high GSDME expression, which causes pyroptosis instead of apoptosis. However, apoptosis occurs in cancer cells with low GSDME levels (14, 15). Hence, the high level of GSDME has the function of converting apoptosis to pyroptosis upon internal and external death stimuli. A commonly used inhibitor of protein palmitoylation, 2-bromopalmitate (2-BP), can affect the localization and function of proteins on the membrane structures (45). A recent study found that 2-BP can inhibit the GSDME-CT palmitoylation and cause a switch from pyroptosis to apoptosis upon treatment with chemotherapeutic agents, but the detailed mechanism needs to be further studied (46).

### 2.4 The granzyme-mediated pathway

Granzyme is an exogenous serine protease derived from cytoplasm particles released by cytotoxic lymphocytes (CTLs) and natural killer cells (NK) and has pro-apoptotic functions (47, 48). Recently, studies have found that granzyme proteases, such as GzmA and GzmB, can induce pyroptosis of cancer cells by cleaving GSDM family members (16, 17). For example, chimeric antigen receptor (CAR) T cells from NK cells and CD8+ T lymphocytes released GzmB into tumor cells in a perforin (PFN)-dependent manner, and then GzmB directly processed GSDME after D270 residue, a site also cleaved by caspase-3, releasing cytotoxic GSDME-NT to form pores in plasmalemma (16). Additionally, it has been demonstrated that NK and CD8+ T cells can induce pyroptosis in cancer cells that express GSDMB to promote tumor clearance *via* the GzmA-GSDMB axis, moreover, this process is enhanced by interferon-γ (IFN-γ) and tumor necrosis factor (TNF). This study demonstrated the idea that the pyroptosis process does not involve caspase activation for the first time (17). More importantly, it was found that cancer cell pyroptosis under the action of Gmzs may amplify inflammation in tumor microenvironments (TMEs), thus recruiting more immune cells and further promoting antitumor immunity and suppressing tumor growth (49). However, cytokine release syndrome (CRS) can be developed in this process, which is a serious and common side effect observed during CAR T-cell therapy (49, 50).

### 2.5 Alternative pyroptosis pathways

Up to now, several alternative pathways have also been elucidated in addition to the above-mentioned pyroptotic pathways. For example, under hypoxic conditions, TNF-α binds to the tumor necrosis factor receptor (TNFR) and subsequently activates caspase-8 expression. When the expression of GSDMC is upregulated in cells through the programmed death ligand 1 (PD-L1)/phospho-signaling transducer and activator of transcription (pSTAT3) pathway transcriptionally, then caspase-8 specifically cleaves GSDMC at Asp240 to form GSDMC-CT and eventually stimulates pyroptosis (51). More recently, Liu Xing et al. reported that streptococcal pyrogenic exotoxin B (SpeB) triggers keratinocyte pyroptosis and further suppresses systemic infection by cleaving GSDMA after Gln246, releasing an active N-terminal fragment to form a GSDMA pore and ultimately triggering pyroptosis (18).

## 3 Pyroptosis in NSCLC-associated signaling pathways

### 3.1 GSDMs

Currently, the human GSDMs superfamily consists of GSDMA, GSDMB, GSDMC, GSDMD, GSDME, and DFNB59 (52). The activating enzymes and biological functions of GSDMD, GSDME, and GSDMC in NSCLC have only recently been made known. GSDMD was first identified as having the function of inducing pyroptosis. Gao et al. for the first time demonstrated the biological effects of GSDMD in NSCLC, and their clinical data showed LUAD and LUSC specimens had significantly higher GSDMD protein levels than matched neighboring tumor specimens (53). In addition, NSCLC patients with larger tumor size and more advanced tumor lymph node metastasis (TNM) stage occurred in patients with GSDMD overexpression. It is found that GSDMD overexpression is an independent poor prognostic biomarker in LUAD, but not in LUSC. Silencing GSDMD expression induced apoptosis, instead of pyroptosis by activating both NLRP3/caspase−1 and caspase−3−mediated mitochondrial pathways *in vitro* and *in vivo*. Meanwhile, knockdown of GSDMD in NSCLC cells were found to undergo apoptosis in the absence of exogenous stimuli, whereas GSDMD-deficient immune cells suffered from apoptosis in the presence of pyroptotic stimuli such as LPS and adenosine triphosphate (ATP). In addition, the epidermal growth factor receptor (EGFR)/protein kinase B (Akt) signal transduction was modulated by GSDMD in NSCLC cell lines. A weakened inflammatory response in GSDMD−deficient NSCLC cells may interrupt the negative feedback regulation that activates the phosphoinositide-3-kinase (PI3K)/Akt pathway. Full-length GSDMD may also play a role in regulating this pathway, but the more nuanced crosstalk between the GSDMD and EGFR/Akt in NSCLC requires further study (53). Xi et al. found that among the GSDM family, only GSDMD gene expression was positively correlated with CD8+ T cell marker genes in cytotoxic T lymphocytes (CTLs) across LUAD, LUSC, and melanoma tumor samples in The Cancer Genome Atlas (TCGA) cohorts, suggesting that the function of GSDMD gene could be related with CD8+ T cells. Furthermore, GSDMD was found to colocalize with GzmB near immune synapses and was essential for an optimal response of CTL to lung cancer cells (54). In addition to GSDMD, the mechanism of GSDME-mediated NSCLC pyroptosis was reported. Compared to paired normal lung tissues, the expressions of GSDME, caspase-3, and caspase-8 were significantly were increased in most lung cancer tissues, and GSDME was mainly found in the cytoplasm and a small amount in the cell membrane in lung cancer tissues. However, the level of GSDME was positively correlated with the postoperative survival rate of patients with lung cancer (55). Similarly, GSDME was modestly upregulated in EGFR-mutant, and downregulation in serine/threonine kinase 11 (STK11)- or Kelch-like ECH-associated protein 1 (KEAP1)/Nuclear factor (erythroid-derived 2)-like 2 (NFE2L2)-mutant in LUAD patients based on the TCGA dataset. Loss of GSDME or caspase-3 significantly attenuated GSDME-dependent pyroptosis in A549, PC9, or NCI-H3122 cancer cells (56). Although the correlation between decreased GSDME levels and tumor size, clinical stage, age, or tumor recurrence rate in NSCLC patients was no significance, however, NSCLC patients with lower GSDME expression had shorter survival times, higher mortality rate, and lower degree of T-cell infiltration after platinum treatment (57). Additionally, Zhang et al. found that both chemotherapeutics paclitaxel and cisplatin (DDP) could induce pyroptosis in A549 cells, the pyroptosis induction by DDP was more severe than that caused by paclitaxel, suggesting that DDP may be more preferable than paclitaxel for the treatment of NSCLC with high GSDME expression (58). It has also been demonstrated that targeted antitumor agents can induce pyroptosis of NSCLC cells by activating the mitochondrial apoptosis-GSDME signaling pathway. GSDME overexpression tended to promote targeted antitumor agent and chemotherapy drug sensitivity to regress NSCLC (56). Dasatinib at low concentrations (0.2 µM) increased GSDME and GSDMD protein levels in A549 cells and further induced pyroptosis, nonetheless, 10 µM dasatinib failed to induce cleavage of GSDME in A549 cells (59). The expression of GSDMC is associated with various cancer (60). It was found that GSDMC is up-regulated in both LUAD tissues and cell lines, especially in radio-resistant LUAD cells. A high level of GSDMC suggested a poor prognosis in LUAD (61). Mechanistically, the expression of GSDMC in LUAD cells may be regulated by DNA hypomethylation (61).

### 3.2 Toll-like receptor 4/NOD-like receptor protein 3

Toll-like receptor (TLRs), a type of pattern recognition receptor (PRRs), plays an important role in multiple diseases (62). It is known that TLR4 can activate the NLRP3 inflammasome and further increase the release of IL-1β and IL-18 (63, 64). Recent investigations have identified that compared with matched normal tissues and human immortalized lung epithelial cell line, the levels of NLRP3, caspase-1, IL-1β, and IL-18 in NSCLC tissues and cells are lower. Additionally, knockdown or reduction of NLRP3 expression significantly promoted NSCLC cell viability and decreased the IL-1β and IL-18 expression, suggesting that NLRP3-caspase-1-IL-1β and IL-18 inflammation pathways play an important antitumor role in NSCLC (65, 66). Meanwhile, Yuan et al. demonstrated that silencing or decreasing TLR4 expression inhibits NLRP3 inflammasome activation and reduces reactive oxygen species (ROS) and calcium ion levels, thus suppressing pyroptosis in NSCLC cells. It was also observed that inhibition of TLR4 by cucurbitacin B treatment suppressed NSCLC growth *via* pyroptosis in a dose-independent manner (67). As a result, the TLR4-mediated NLRP3 signaling pathway is a potential target for NSCLC therapy.

### 3.3 ROS/Nuclear factor-κB

Oxidative stress is characterized by a biological state of imbalance between the production and elimination of ROS, which is mainly produced by damaged mitochondria (68, 69). The excessive level of ROS can activate NF-κB, leading to a persistent and strong inflammatory response (70). ROS has also been considered to be one of the exciters that activate NLRP3 inflammasome (71). NF-κB is abnormally activated in NSCLC tissues and cells, and an increase of in NF-κB can promote NSCLC cells pyroptosis (72, 73). Polyphyllin V was recently found to induce pyroptosis in NSCLC cells through the stimulation of the ROS/NF-κB/NLRP3/GSDMD axis. N-acetyl-L-cysteine (NAC) has the potential to inhibit NF-κB and NLRP3 expression and pyroptosis (73). Conversely, ROS has been shown to induce pyroptosis by inhibiting NF‐κB signaling (74). Li et al. found that increasing ROS levels in NSCLC cells with a small molecule ROS inducer can induce apoptosis and pyroptosis, and ROS-mediated NF-κB inhibition may be one of the mechanisms of pyroptosis (75). Meanwhile, Chen et al. also reported that NF-κB system suppression exhibited good antitumor activity in NSCLC cells by switching apoptosis to pyroptosis *in vitro* and *in vivo* (76, 77).

### 3.4 Long noncoding RNAs and microRNAs

The development of numerous cancers, including NSCLC, and the emergence of drug resistance was strongly correlated with lncRNAs and miRNAs (78, 79). According to recent studies, lncRNA X inactive-specific transcript (XIST) was aberrantly overexpressed in NSCLC tissues and cell lines and was especially in NSCLC tumors treated with (DDP) (80, 81). Knock-down of lncRNA-XIST promoted NSCLC cell apoptosis and pyroptosis (80, 81). Liu et al. found that lncRNA-XIST knockdown induced NLRP3 inflammasome activation and affected ROS-induced pyroptotic cell death by regulating miRNA-335/superoxide dismutase 2 (SOD2) in A549 cells (80). Moreover, the sensitivity of NSCLC cells to DDP increased after lncrNA-XIST knockout. Additionally, the carcinogenesis roles and abilities to promote DDP resistance of lncRNA-XIST were largely associated with the reduction of nuclear translocation of TGF-β effector mothers against decapentaplegic homolog 2 (SMAD2), which further prevented the transcription of p53 and NLRP3 (81). Song et al. also demonstrated several lncRNAs are associated with pyroptosis, and generated a new risk signal for predicting LUAD prognosis, providing new prospects for promoting personalized treatment for LUAD patients (82). Shi et al. showed miRNA-556-5p, a newly discovered pyroptosis-related gene that regulates DDP-chemoresistance in NSCLC. They found that miRNA-556-5p level was obviously up-regulated in the DDP-chemoresistance NSCLC tissues and cells. Further experiments confirmed that knocking out miRNA-556-5p suppresses NSCLC cell viability by up-regulating NLRP-mediated pyroptotic cell death. Interestingly, the pyroptotic effects of miRNA-556-5p silencing were reversed by necrosulfonamide (NSA) and NLRP3 reduction, suggesting that the miRNA-556-5p/NLRP3 pathway can regulate DDP-chemoresistance in NSCLC (83).

### 3.5 Protein 53

The tumor suppressor gene p53 is a major transcription factor, and activated p53 can inhibit tumorigenesis by regulating the transcription of a series of genes. Mutant p53 has no tumor-suppressive activity, but instead acquires a dominant-negative effect and novel tumorigenic functions (84). NSCLC occurrence and progression are closely correlated with p53 (85). Mounting evidence suggests that p53 also inhibits NSLCLC tumors by promoting pyroptosis. Zhang et al. for the first time revealed that p53 positively correlates with pyroptosis in NSCLC tumor tissues (86). According to their findings, overexpression of p53 induced pyroptosis and the mRNA and protein levels of NLRP3, ASC, and cleaved caspase-1 were all increased. Based on chromatin immunoprecipitation (ChIP) analysis, they found that P53 can interact directly with NLRP3. Furthermore, high expression of p53 markedly inhibited NSCLC growth by inducing pyroptosis, suggesting that p53 can be considered a potent regulator capable of mediating pyroptosis in NSCLC effectively (86).

### 3.6 Maternal embryonic leucine zipper kinase

MELK is a fascinating cell cycle-dependent protein kinase belonging to the sucrose non-fermenting 1 (Snf1)/adenosine monophosphate-activated kinase (AMPK) family and is highly expressed in most malignant tumors (87). MELK overexpression is related to poor prognosis of cancer. MELK is implicated in tumor cell cycle regulation, proliferation, apoptosis, invasion, and metastasis (87). Lai et al. found that high MELK expression was negatively correlated with LUAD survival. The migration and invasion activities of LUAD cells was inhibited after knockdown of MELK expression by decreasing the levels of twist family BHLH transcription factor 1 (Twist1), snail family transcriptional repressor 2 (Snai2), matrix metalloproteinase 7 (MMP7), and N-catenin. In terms of the molecular mechanism of MELK in LUAD, inhibition of MELK by the specific inhibitor of MELK OTSSP167 arrested the G2/M phase of LUAD cells *via* the polo-like kinase 1 (PLK-1)/cell division cycle (CDC25C)/cyclin-dependent kinase 1 (CDK1) pathway and triggered the caspase-3/GSDME-mediated pyroptosis. Therefore, MELK may be a promising biological target for LUAD in clinic in the future (88).

### 3.7 Apurinic/apyrimidinic endonuclease 1

APE1, a biological enzyme with multiple functions, mainly primarily stimulates several transcription factors in DNA repair and redox. APE1 also plays a part in genomic DNA oxidation and alkylation base repair by recognizing and cutting nucleotide chains at the 5’-apurinic (AP) site during the DNA base excision repair (BER) process (89, 90). Recent studies have found that APE1 is highly expressed in a variety of cancers, and knockdown of MELK expression decreases cancer cell proliferation and induces apoptosis (91). Long et al. concluded that APE1 is a poor prognosis marker in NSCLC, and the suppression of APE1 with the APE1 inhibitor NO.0449-0145 could promote the accumulation of unrepaired DNA damage in NSCLC cells continuously and induce DNA damage, apoptosis, pyroptosis, and necroptosis *in vitro* and *in vivo*, as well as overcome both DDP- and erlotinib-resistance in NSCLC. In terms of the pyroptosis mechanism, APE1 downregulation induced by NO.0449-0145 can upregulate the expression of caspase-4, GSDMD-NT, and IL-1β (92).

### 3.8 Secretoglobin family 3A member 2

SCGB3A2 (also named uteroglobin-related protein 1, UGRP1 and high in normal-2, HIN-2), the first member of the SCGB gene superfamily, that is highly expressed in airway epithelial Club cells with various biological functions, including anti-lung cancer activity (93–95). Yokoyama et al. found that SCGB3A2 chaperones LPS to the cytosol through the heparan sulfate (HS) of the cell surface receptor syndecan-1 (SDC1) and activates the caspase-11/NLRP3/GSDMD pathway to lead to pyroptosis in murine Lewis lung carcinoma (LLC) cells (96). Recent studies further show that SCGB3A2 exhibits marked anticancer activity against 5 out of 11 human NSCLC cell lines, while no effect on small cell lung cancer cell lines. Interestingly, better survival outcomes were observed in patients with LUAD having high SCGB3A2 expression. Moreover, it was found that SCGB3A2 and LPS together induce growth inhibition of NCI-H596 cancer cells through caspase-4/GSDMD-mediated pyroptosis (97). Therefore, SCGB3A2 uses the machinery of pyroptosis for the elimination of human NSCLC *via* the non-canonical inflammasome pathway.

## 4 The role of pyroptosis in NSCLC therapy

### 4.1 Pyroptosis is a potent aide in chemotherapy, targeted therapy, and immunotherapy

#### 4.1.1 Chemotherapy

Paclitaxel and DDP are first-line chemotherapy drugs used for a variety of other malignancies including advanced NSCLC (98, 99). Mechanicall, paclitaxel and DDP lead to tumor cell apoptosis mainly by targeting microtubules and DNA, respectively (100). Recent studies have shown that in addition to apoptosis, paclitaxel and DDP can trigger A549 cells pyroptosis *via* the caspase-3/GSDME pathway, with DDP triggering more pronounced pyroptosis as well as higher levels of caspase-3 and GSDME-NT than paclitaxel (58). Nevertheless, chemotherapy regimens cannot effectively improve the overall survival (OS) of NSCLC because apparent apoptotic resistance is common in these patients (2). Therefore, other types of non-apoptotic programmed cell death, such as pyroptosis, should be explored to enhance chemotherapy sensitivity. A recent investigation has suggested that GSDME-induced pyroptosis can increase the chemotherapy sensitivity of NSCLC cells to DDP. The study further suggested that GSDME silencing can reverse DDP-induced NSCLC growth inhibition rather than tumor regression *in vivo* (57). Natural products, repurposed drugs, small molecule compounds, and conventional chemotherapeutic agents have all been found to induce pyroptosis in NSCLC. For example, doxycycline and simvastatin are originally broad-spectrum tetracycline-class antibiotics and antilipemic agents, respectively (65, 101). As studies progressed, it became clear that both repurposed drugs had strong anticancer effects on NSCLC *via* the NLRP3-caspase-1-mediated pyroptosis pathway. Wu et al. reported three synthetic small molecule NF-κB inhibitors that exhibit good antitumor activity by switching apoptosis to pyroptosis (75–77) A small molecule compound NO.0449-0145 was also discovered as an APE1 inhibitor through high-throughput virtual screening of small molecule libraries by Long et al. Furthermore, the level of caspase-4 and GSDMD *in vitro* and *in vivo* was upregulated following NO.0449-0145 treatment (92). Due to excellent anticancer and safety properties, natural products are considered another promising alternative for first-line cancer prevention and treatment (102). Polyphyllin VI is a natural product that induces pyroptosis through the ROS/NF-κB/NLRP3/GSDMD signaling axis in NSCLC, suggesting that polyphyllin VI is a potential therapeutic option for NSCLC patients (73). Cucurbitacin B (CuB) is a natural tetracyclic triterpenoid derived from cucurbitaceae and cruciferous plants. Recent study showed that CuB is an effective pyroptosis inducer in NSCLC *in vivo* and *in vitro* by stimulating the TLR4/NLRP3/GSDMD-dependent signal pathway (67). Cordyceps militaris extract (CME) was also found to induce apoptosis and pyroptosis *via* the caspase-3/poly (ADP-ribose) (PARP)/GSDME pathway in the A549 cells, providing fundamental insights into the clinical application of CME in patients with NSCLC (103).

#### 4.1.2 Targeted therapy

Molecularly targeted therapies are one of the main effective strategies for stimulating apoptosis machinery in the treatment of NSCLC (104). Compared with traditional chemotherapy, molecularly targeted therapy drugs can kill tumor cells in a targeted manner with mild side effects and high safety (104). However, resistance to molecularly targeted therapies often leads to poor anticancer outcomes and even therapeutic failure (2). Lu et al. concluded that GSDME-dependent cell pyroptosis is another widespread cancer death mode, and GSDME overexpression promotes small molecular targeted inhibitor sensitivity. This result suggest that pyroptosis may serve as an ally to improve the antitumor efficacy of targeted drugs in NSCLC. Additionally, they found that several kirsten rat sarcoma virus (KRAS), the epidermal growth factor receptor (EGFR), or anaplastic lymphoma kinase (ALK) small molecule targeted inhibitors uniformly activate the mitochondrial intrinsic apoptotic pathway to facilitate caspase-3 cleavage of GSDME to induce NSCLC cell pyroptosis, in addition, to concurrently inducing cellular apoptosis. The results of this study change the traditional viewpoint that that molecular targeted therapy can only induce cell death through the apoptotic pathway, established the clinical biological correlation between GSDME expression and pyroptosis, and propose that pyroptosis can improve the sensitivity and antitumor efficacy of targeted drugs, although these effects are decreased when apoptotic feature is unabridged (56).

#### 4.1.3 Immunotherapy

Unlike non-inflammatory apoptosis, pyrotopia is a type of cell death that induces a strong inflammatory response and in some cases is considered an immunogenic cell death (30). Although many researches are needed to clarify the relationship between pyroptosis and anticancer immunity currently, several studies suggest that pyroptosis exerts antitumor effects by enhancing immune activation and function in NSCLC. For example, CTLs are able to release the contents of potentially anti-immune cytotoxic particles into the immune synapse formed with target cancer cells (105). Moreover, CTLs can release perforin and Gzms onto target cells and then promote pore formation and cell lysis. These morphological changes are similar to pyroptosis (47). Xi et al. showed that CTL with higher expression of GSDMD had stronger inhibition effects on lung cancer cells. CTL-mediated pyroptosis is partly *via* activation of caspase-4. They observed that silencing caspase-4 reversed CTL activation and GSDMD-mediated pyroptosis in NSCLC cell line H1299. Furthermore, CTL cytotoxicity towards H1299 cells and human CTLs was diminished following GSDMD knockdown, indicating that GSDMD-induced pyroptosis was required for CD8+ T cell cytotoxicity (54). Peng et al. also reported that the cell survival rate of GSDME-silenced LLC tumor was elevated after DDP treatment in nude mice, while a prominent reduction in tumor volume was observed in GSDME-overexpressing LLC xenograft after DDP treatment in C57 mice. Because nude mice lack a thymus and C57 mice have a normal immune function, the results suggested that GSDME is involved in the antitumor therapy *via* immune regulation. Further study found that the number of tumor-infiltrating CD3+ T cells was enhanced when increased GSDME expression and the levels of chemokines that recruit T-cells after DDP treatment, indicating that GSDME may induce T-cell activation in mediating T-cell infiltration into tumor tissue (57). Although anticancer immunotherapeutic strategies have demonstrated efficacy against lymphoid tumors, they are less effective against solid tumors owing to the presence of an immunosuppressive microenvironment induced through programmed death-1 (PD-1) (106). To interfere with PD-1 signaling to enhance immunotherapy for solid tumors, NK92 cells inspired a chimeric costimulatory converting receptor (CCCR) (CCCR-NK92) was established by Lu et al., and these CCCR-NK92 cells can reversed the immune suppressive effects of PD-1 by switching the negative PD-1 signal to an activating signal, and hence effectively enhanced the antitumor activity against H1299 cancer cells by triggering GSDME-induced pyroptosis (107). In addition, CCCR-NK92 cells significantly inhibited tumor growth *in vivo*, suggesting this CCCR-NK92 cells may provide an effective option for promoting NSCLC immunotherapy (107).

### 4.2 The prognostic role of pyroptosis-related biomarkers in NSCLC

#### 4.2.1 The prognostic value and mechanism of GSDMs in NSCLC

The GSDMD family, especially GSDMC, GSDMD, and GSDME can be consider as potential markers for the diagnosis and prognosis of lung cancer patients, including NSCLC. Dysregulated GSDMC is associated with a variety of cancers and its functions are tissue-specific (60). GSDMC is found to be significantly overexpressed in LUAD tissues and cell lines compared to adjacent normal tissues, suggesting that GSDMC is a promising promoting cancer biomarker in LUAD []61. Moreover, LUAD patients with lower GSDMC levels showed preferable first progression and OS, and GSDMC expression in LUAD cells may be regulated by DNA hypomethylation (61). DNA hypomethylation plays an important role in regulating the transcription of important oncogenes in various cancers including LUAD (108). The preferentially expressed phosphoribosylaminoimidazole carboxylase (PAICS) is the common tumor promoter in LUAD and can be activated by promoter DNA hypomethylation. Tumor suppression caused by overexpression of adenosine deaminase RNA-specific B1 (ADARB1) is the result of DNA demethylation signal transduction, and inactivation of DNA methylation using a DNA methyltransferase inhibitor significantly enhances ADARB1-mediated metastatic inhibition of LUAD cells (109). In a study by Wei et al., GSDMC was hypomethylated in the lung tissue of patients with LUAD compared with normal lung tissue, and in all cases, GSDMC expression was significantly negatively correlated with its methylation status (61). The expression of GSDMC was negatively correlated with the methylation degree of two CpG sites (cg05316065 and cg26073844) in its promoter region (61). The longer the OS of patients with LUAD, the higher the degree of GSDMC methylation. These suggest that DNA hypomethylation plays an important role in the overexpression of GSDMC, and up-regulated GSDMC may serve as an independent predictor of poor prognosis in patients with LUAD (61). The level of GSDMD protein in lung cancer was significantly upregulated, and the expression of GSDMD was not correlated with age, gender, and lymph node metastasis in patients with LUAD; high expression of GSDMD was significantly correlated with tumor size and later TNM staging in LUAD patients. However, the expression of GSDMD was only significantly correlated with the patient’s TNM stage in LUSC, and the inconsistent results may be due to differences in histological subtypes of the tumor (53). The above results indicated that GSDMD can be regard as an independent prognostic biomarker in LUAD patients. Meanwhile, the expression level of GSDME in lung cancer tissue is significantly higher than that in adjacent tissue, patients with lung cancer having a high GSDME expression have less lymph node metastasis and longer postoperative survival, although the decrease in GSDME level was not significantly correlated with tumor size, clinical stage, age, or tumor recurrence rate (55, 110). In addition, the expression level of GSDME was positively correlated with that of caspase-3, and hence caspase-3 is also an important prognostic biomarker in lung cancer patients (55).

#### 4.2.2 The prognostic value and mechanism of other pyroptosis-related genes in NSCLC

Mounting evidence indicates that abnormally expressed lncRNA has prognostic value in a variety of biological and pathological processes. Song et al. discovered that five pyroptosis-related genes, including lncRNAs-GSEC, FAM83A-AS1, AL606489.1, AL034397.3, and AC010980.2, were significantly associated with OS in LUAD patients. Among these genes, GSEC, FAM83A-AS1, AL606489.1, and AC010980.2 are considered as potentially dangerous lncRNAs, while AL034397.3 is a defensive lncRNA (111). Moreover, an immune-related risk model was established using AL034397.3 showed that AL034397.3 is a reliable predictive marker of LUAD prognosis. Other studies have also confirmed that immunity, autophagy, and ferroptosis are closely related to AC010980.2 in LUAD. AC010980.2 could be a carcinogene to predict the prognosis of LUAD patients (112, 113). The study by Lin et al. found novel pyroptosis-related prognostic genes in patients with LUAD including NLRP7, NLRP1, NLRP2, NOD1, and caspase-6 (114). Prognostic analysis showed patients with low expressions of NLRP7, NLRP1, NLRP2, and NOD1 and high expression of caspase-6 always have poor survival, respectively. Moreover, the above five pyroptosis-related prognostic biomarkers are closely related to immune infiltration, indicating that pyroptosis can exert antitumor efficacy by mediating LUAD in the tumor immune microenvironment. Also, miRNA-335-5p and lncRNA KCNQ1OT1 are related to the prognosis of LUAD patients, and further study showed that the lncRNA KCNQ1OT1/miRNA-335-5p/NLRP1/NLRP7 regulatory axis may play a crucial role in the progression of LUAD (114). Additionally, IL-6, NOD1, and caspase-4 are pivot genes and could be used as potential prognostic factors for early-stage LUSC. Among these, IL-6 has been established as a potent independent risk factor for OS in early-stage LUSC patients, and patients with higher levels of IL-6 expression tend to have advanced LUSC and large cell lung cancer (115). NOD1 may be an antioncogene of lung cancer, and high expression of caspase-4 is related to poorer survival outcomes, possibly due to the negative regulation of pyroptosis by caspase-4.

The lack of comprehensive and personalized treatment is one of the reasons for the high mortality rate associated with NSCLC. Therefore, identifying new genes for early prognostic detection may be of great significance for discovering new therapeutic targets for NSCLC. The above studies have shown that the expression of pyroptosis-related genes can affect the prognosis of patients with NSCLC. Compared with the TNM staging system, pyroptosis-related genes may be used as markers of lung cancer prognosis in the future.

## 5 Future perspective and conclusion

In this review, we described for the first time the relationship between the cellular physiology and molecular mechanisms of pyroptosis and NSCLC (**Figures 2** and **3**). Pyroptosis is a defense mechanism against pathogenic insults that has recently been linked to GSDMs-dependent programmed death, which is distinct from apoptosis, necrosis, autophagy, and ferroptosis and has a unique mode of occurrence. Although the underlying mechanism that regulates pyroptosis has not been fully revealed, it has been implicated in the regulation of multiple signaling pathways and intracellular biological processes in NSCLC progression. Numerous studies have shown that NSCLC cell lines exhibit severe chemoresistance due to resistance to apoptosis, and therefore the induction of pyroptosis appears promising for NSCLC treatment. It has also been indicated that the pyroptosis-based therapeutic strategies, including chemotherapy, targeted therapy, and immunotherapy may be able to inhibit the progression of NSCLC *in vitro* or *in vivo* and target pyroptosis, enhancing anti-NSCLC cancer efficacy (**Table 1**). Meanwhile, various pyroptosis-related genes with great clinical significance were identified, and they play important roles in tumor immunity and can be used to predict the prognosis of LUAD and LUSC, but the scope of bioinformatics analysis should be expanded, and relevant biological experiments at the cell, animal, and tissue level should be performed to further confirm the scientificity of these genes.

**Table 1:** The mechanism of pyroptosis in NSCLC.

Author year	Cell/Animal model/Human tissue	Mechanism	Ref
Gao et al., 2018	PC9, H1703 and H1975/PC9 mouse xenograft model/LUAD, LUSC tissue	Knockdown of GSDMD restricted NSCLC growth by promoting apoptosis and inhibiting EGFR/Akt signaling	(53)
Xi et al., 2019	H1299	GSDMD deficiency reduced the cytolytic capacity of CD8+ T cells to H1299 cells	(54)
Lu et al., 2018	PC9, A549 and H3122/H3122, HCC827 mouse xenograft model/lung cancer tissue	Molecular Targeted drugs indued GSDME-dependent pyroptotic tumor cell death	(56)
Zhang et al., 2020	A549	Low concentrations of dasatinib can induced cell pyroptosis by increasing GSDMD and GSDME levels	(59)
Zhang et al., 2019	A549	paclitaxel and cisplatin differentially induce pyroptosis in A549 lung cancer cells *via* caspase-3/GSDME activation	(58)
Huang et al., 2020	Lung cancer tissue	High GSDME expression experienced statistically fewer lymph node metastasis and had a higher prognostic survival rate	(55)
Peng et al., 2020	A549, H1299/LLC, A549 and H1299 mouse xenograft model/Lung cancer tissue	GSDME-mediated pyroptosis provides the antitumor role of DDP treatment by releasing chemokines to recruit T cell	(57)
Wei et al., 2020	LUAD tissue	Upregulated GSDMC expression was independent indicator of poor first progression (FP) and overall survival (OS) in LUAD patients	(60)
Xie et al., 2019	A549, H520 and H358/H520 mouse xenograft model/NSCLC tissue	Huaier extract exhibited antitumor effect in NSCLC *via* inducing NLRP3-dependent pyroptosis	(66)
Wang et al., 2018	A549, H1299/H1299 mouse xenograft model/NSCLC tissue	Simvastatin induced pyroptosis in NSCLC by activating NLRP3 pathway	(65)
Yuan et al., 2021	A549, H1299/LLC mouse xenograft model	Cucurbitacin B inhibited NSCLC by triggering TLR4/NLRP3/GSDMD-dependent pyroptosis	(67)
Teng et al., 2021	A549, H1299	Polyphyllin VI induced Caspase-1-mediated pyroptosis *via* triggering ROS/NF-κB/NLRP3/GSDMD pathway in NSCLC	(73)
Chen et al., 2018	H460/H460 mouse xenograft model	L61H10 induced apoptosis and pyroptosis *via* NF−κB pathway	(77)
Chen et al., 2019	H460/H460 mouse xenograft model	13d induced pyroptosis developing from apoptosis *via* the inhibition of NF‐κB	(76)
Li et al., 2019	A549	L50377 induced pyroptosis through ROS mediated NF-κB inhibition	(75)
Liu et al., 2019	A549, H1299/NSCLC tissue	Knockdown of LncRNA-XIST induced NSCLC pyroptosis by triggering miR-335/SOD2/ROS pathway	(80)
Xu et al., 2020	A549, H1299, A549/DDP, H1299/DDP/NSCLC tissue	LncRNA-XIST knockdown induced NSCLC pyroptosis and promoted DDP chemosensitivity through SMAD2/NLRP3 pathway	(81)
Shi et al., 2021	A549, H1299, A549/DDP, H1299/DDP/NSCLC tissue	MiR-556-5p ablation induced NSCLC pyroptosis and promoted DDP chemosensitivity through *via* upregulating NLRP3	(83)
Zhang et al., 2019	A549/NSCLC tissue	Upregulation of p53 prompts pyroptosis to produce anti-NSCLC effects	(86)
Tang et al., 2019	95-D, H1299 and H1975/95-D_NC, 95 D_MELK, H1299_NC and H1299_shMELK mouse xenograft model/LUAD tissues	Inhibition of MELK triggered apoptosis-mediated pyroptosis	(88)
Long et al., 2021	A549, H460, H1299, H1975-ER and A549-DDP/H460 mouse xenograft model/NSCLC tissue	Inhibition of APE1 can induce pyroptosis and overcame both cisplatin- and erlotinib-resistance in NSCLC	(92)
Yokoyama et al., 2021	H596/H596, H358, H157 mouse xenograft model	SCGB3A2 inhibit NSCLC growth/metastasis by means of the Caspse-4-mediated pyroptosis pathway	(97)

However, there are still several pressing issues that require further investigation. As an inflammatory cell death mode, the fatal cytokine release syndrome (CRS) could be occurred during pyroptosis therapy, which damages adjacent or other normal tissues and further leads to a series of adverse effects. In other words, the improper control of pyroptosis cannot provide benefits to patients. How to regulate the pyroptosis of tumor cells to achieve the desired therapeutic effect while avoiding the necrosis of normal cells remains to be explored. Perhaps targeting the pyroptosis of tumor cells could become a promising anticancer therapy approach in the future. Targeted inhibitors were found to induce NSCLC cell death by inducing pyroptosis, indicating the therapeutic possibility of targeting pyroptosis. Moreover, pyroptosis induction is strongly associated with carcinogenesis effects and a poor prognosis. Meanwhile, the activation of both GSDMD and GSDME can trigger pyroptosis in NSCLC. Whether the activation of GSDMD or GSDME is more beneficial is currently unclear and further research is needed.

## Author contributions

Study concept, design and drafting of the manuscript: JBW and JZW. Write the manuscript: XC. All authors have read and agreed to the published version of the manuscript. All authors contributed to the article and approved the submitted version.

## Funding

The work was supported by the National Natural Science Foundation of China (No. 81903074), the Medical Health Science and Technology Project of Zhejiang Provincial Health Commission (No. 2020KY366 and 2021KY399).

## Conflict of interest

The authors declare that the research was conducted in the absence of any commercial or financial relationships that could be construed as a potential conflict of interest.

## Publisher’s note

All claims expressed in this article are solely those of the authors and do not necessarily represent those of their affiliated organizations, or those of the publisher, the editors and the reviewers. Any product that may be evaluated in this article, or claim that may be made by its manufacturer, is not guaranteed or endorsed by the publisher.
